# Crack Resistance of Lightly Reinforced Concrete Structures

**DOI:** 10.3390/ma17102197

**Published:** 2024-05-08

**Authors:** Marta Słowik, Ewa Błazik-Borowa, Maria Jolanta Sulewska, Izabela Skrzypczak, Wanda Kokoszka

**Affiliations:** 1 Faculty of Civil Engineering and Architecture, Lublin University of Technology, Nadbystrzycka 38D, 20-618 Lublin, Poland; e.blazik@pollub.pl; 2 Faculty of Civil Engineering and Environmental Sciences, Bialystok University of Technology, Wiejska 45E, 15-351 Bialystok, Poland; m.sulewska@pb.edu.pl; 3 Faculty of Civil and Environmental Engineering and Architecture, Rzeszow University of Technology, Powstancow Warszawy 12, 35-959 Rzeszow, Poland; izas@prz.edu.pl (I.S.); wandak@prz.edu.pl (W.K.)

**Keywords:** concrete structures, crack resistance, reinforcement ratio, flexural concrete members, scale effect, fracture parameters of concrete

## Abstract

The crack resistance of concrete structures with low reinforcement ratios requires a broader examination. It is particularly important in the case of foundations working in changing subsoil conditions. Unfavorable phenomena occurring in the subsoil (e.g., ground subsidence, landslips, non-uniform settlement) can lead to unexpected cracking. Therefore, it is necessary to check the effectiveness of the low reinforcement provided. As there are limited studies on lightly reinforced concrete structures, we performed our own experimental investigation and numerical calculations. In the beams analyzed, the reinforcement ratio varied from 0.05% to 0.20%. It was found that crack resistance in concrete members depends on the reinforcement ratio and steel bar distribution. A comprehensive method was proposed for estimating the crack resistance of lightly reinforced concrete members in which both the reinforcement ratio and the reinforcement dispersion ratio were taken into account. Furthermore, the method considered the size effect and the fracture properties of concrete. The proposed method provides the basis for extrapolation of the test results obtained for small elements and conclusions for members with large cross-sections, such as foundations, which frequently use lightly reinforced concrete.

## 1. Introduction

In typical building structures and dwelling houses, the foundations are often made of low-reinforced concrete or plain concrete. The use of reinforcement, even with a low cross-section, can effectively transfer tensile stresses and protect the foundation against excessive crack width. This is particularly important in the case of foundations exposed to subsoil conditions, which change during the exploitation of a building structure. Changes in the subsoil conditions caused, for example, by non-uniform settlement, landslides or under-washing of soil may lead to a stress state in the foundation, which may not have been taken into account in the design. The basic rules for determining the safety and durability of structural members are specified in EN 1990 [1]. Verification of building structure reliability depends on several factors, among them the quality of materials, unfavorable environmental conditions, the maintenance of exploitation, design working time and applied loads (as described by Skrzypczak, Słowik and Buda-Ożóg in Ref. [2]). However, the primary factor is connected to requirements regarding the member’s capacity. Therefore, a proper estimation of the load-carrying capacity and crack resistance of plain concrete and lightly reinforced concrete members is of paramount importance.

Crack resistance governs the load-carrying capacity of unreinforced concrete members. However, the experimental results from different studies vary significantly. When comparing the results reported in the literature (see Refs. [3,4,5,6,7,8,9,10,11]), a general trend can be observed whereby a decrease in member size results in higher load capacity. The influence of the size effect on crack resistance was the subject of interest for several researchers, for example, Bažant and Pfeiffer [3], Hillerborg, Modeer and Petersson [9], Zhu [10], Chandram and Galyon [11]. It appears that size effect is the main factor influencing the crack resistance of concrete structures.

**Figure 1:**
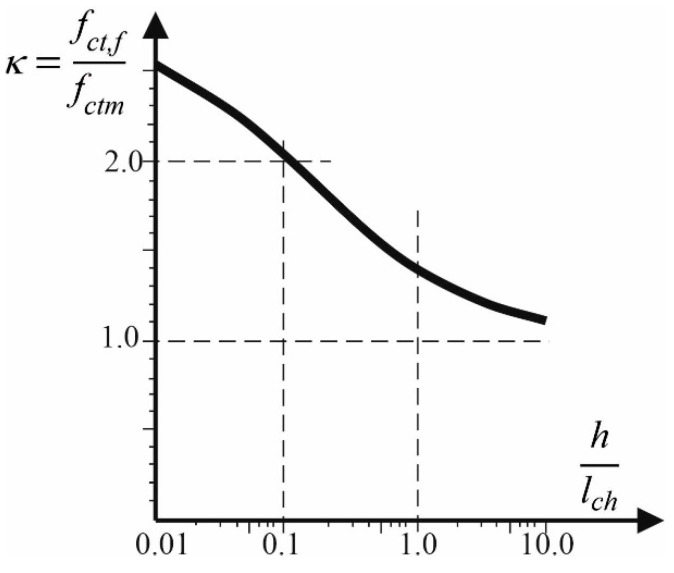
Coefficient *κ,* based on Ref. [9].

When determining the cracking moment in flexural concrete members, the flexural tensile concrete strength *f_ct,f_* should be used. It is higher than the axial tensile concrete strength *f_ctm_* and, as stated in Refs. [3,4,5,6,7,8,9,10,11], the difference can be significant depending on many factors, primarily on the cross-section height of the member. The influence of member’s dimensions on the so-called flexural tensile concrete strength *f_ct,f_* was analyzed by Hillerborg, Modeer and Peterson [9]. The coefficient *κ* was used in Ref. [9] to describe the ratio of flexural tensile concrete strength to the axial concrete strength *κ* = *f_ct,f_*/*f_ctm_*. On the basis of a series of numerical calculations, which were performed using the principles of non-linear fracture mechanics and a non-linear model of concrete in tension, researchers derived the dependence of coefficient *κ* on the ratio of section height *h* to the characteristic concrete length *l_ch_* (shown in Figure 1). The coefficient *κ* depends not only on the cross-section leading to dimension–height *h* but also on the characteristic concrete length *l_ch_*. The characteristic concrete length *l_ch_* is the parameter, which comprehensively describes the main fracture characteristics of concrete, including the fracture energy of concrete *G_F_* and the modulus of elasticity of concrete in tension *E_ct_*, and it is described by the formula

(1) $$l_{ch}=\frac{G_{F}E_{ct}}{f_{ctm}^{2}},$$

where

*l_ch_*—the characteristic concrete length;
*E_ct_*—the modulus of elasticity of concrete;
*G_F_*—the fracture energy of concrete;
*f_ctm_*—the tensile strength of concrete.

The characteristic concrete length *l_ch_* depends on the fracture energy of concrete and thus on the maximum size of aggregate grains *D*_max_ because—as observed during experiments [10]—the aggregate particle size distribution has an impact on fracture energy. The influence of the maximum aggregate size on fracture energy is taken into account in the CEB-FIP Model Code [12], where the following formula for estimating *G_F_* is proposed:

(2) $$G_{F}=\alpha_{F}f_{cm}^{0.7},$$

where *α_F_*—the coefficient, which depends on the maximum aggregate size *D_max_* (*α_F_* = 4 for *D_max_* = 8 mm, *α_F_* = 6 for *D_max_* = 16 mm, *α_F_* =10 for *D_max_* = 32 mm); *f_cm_*—the compressive strength of concrete (mean value of cylindrical compressive strength).

The coefficient *κ* can be used for calculating the crack resistance in concrete members based on the formula

(3) $$M_{cr}=W_{c}f_{ct,f}=W_{c}\kappa f_{ctm},$$

where *W_c_* is the section modulus.

Only a few studies on lightly reinforced concrete structures were found in the scientific literature [13,14,15,16,17]. In France, the load-carrying capacity of beams subjected to bending was studied by Chambaud [13]. In his view, what distinguishes lightly reinforced members is that the stresses in the steel during a member’s failure exceed the yield stress *f_y_* and can reach the value of *σ_s_* = 1.3*f_y_*. This phenomenon was previously described by Saliger [14], who defined it as the self-hardening of steel. In Poland, the load-carrying capacity of lightly reinforced concrete members was investigated by Dąbrowski [15]. On the basis of the experimental investigation performed, an increase in the crack resistance of lightly reinforced concrete beams compared to the cracking moment in members without reinforcement was reported in Ref. [15]. Furthermore, it was noticed that the increase in crack resistance depended on the longitudinal reinforcement ratio *ρ* and was more visible in beams with *ρ* < 0.08% than in beams with *ρ* > 0.08%.

To determine the influence of reinforcement and member’s size on the crack resistance of concrete members with low reinforcement ratios, we conducted our own experimental research. Furthermore, a study was carried out on the mechanism governing crack initiation in a concrete member with different distribution of reinforcing bars. The currently predominant opinion (e.g., in Refs. [18,19,20,21,22,23,24]) is that the best explanation for the fracture behavior of concrete can be formulated by the principles of fracture mechanics. Therefore, a numerical analysis based on non-linear fracture mechanics was performed in order to provide better insight into the phenomena associated with the tensile fracture of concrete in flexural members. The numerical analysis was verified based on our own experimental results.

The application of fiber reinforcement to concrete can effectively increase its crack resistance. A summary of recent scientific papers focusing on the application of fibers in concrete structures was presented in Refs. [25,26]. However, several existing structures made of concrete without fibers as reinforcement require capacity checking over their period of use. Therefore, the investigation performed addressed concrete members with conventional steel bars as reinforcement.

## 2. Materials and Methods

### 2.1. Experimental Investigation

The experimental investigation performed aimed at the determination of the cracking moment of flexural concrete members. Laboratory tests were performed using rectangular cross-section beams with the following dimensions: 0.14 m (width), 0.30 m (height), 3.00 m (length). Three unreinforced concrete beams and ten lightly reinforced concrete beams were tested. Lightly reinforced concrete beams were characterized by different reinforcement ratios: two beams with *ρ* = 0.05%, two beams with *ρ* = 0.07%, two beams with *ρ* = 0.09%, two beams with *ρ* = 0.12%, two beams with *ρ* = 0.20%. The experimental investigation was performed in two stages. In the first stage, unreinforced concrete beams and beams with reinforcement ratios of *ρ* = 0.12% and *ρ* = 0.20% were produced. In the second stage, lightly reinforced beams with reinforcement ratios of *ρ* = 0.05%, *ρ* = 0.07% and *ρ* = 0.90% were produced. Each time the beams were concreted, a concrete mixture was used to prepare the specimens for testing the properties of hardened concrete.

The mechanical properties of concrete were tested using standard methods [27,28,29,30,31]. The compressive strength of concrete was tested on 150 mm cubes. In order to obtain the cylindrical compressive strength *f_c,cyl_*, the cubic compressive strength obtained from the test *f_c,cube_* was recalculated using the relation *f_c,cyl_* = 0.8 *f_c,cube_*. The tensile strength of concrete was tested on 150 mm cubes by performing a splitting tensile test. The axial tensile strength *f_ct,ax_* was estimated from the splitting tensile strength *f_ct,sp_* according to the formula given in the CEB-FIP Model Code [12]: *f_ct,ax_* = 0.9 *f_ct,sp_*. The secant modulus of elasticity was tested on cylinders with a diameter of 150 mm and a height of 300 mm. The final results were statistically worked up, and the mean value of cylindrical compressive strength *f_cm_*, the mean value of axial tensile strength *f_ctm_*, the mean value of modulus of elasticity *E_cm_* and the fracture energy of concrete *G_F_* are listed in Table 1. The fracture energy was estimated using Equation (2).

**Figure 2:**
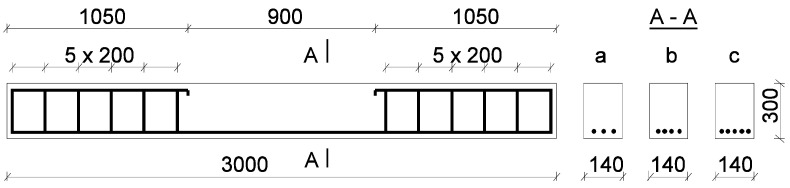
The location of reinforcement in the beam (dimensions in millimeters).

Different steel bars were used in the beams: ϕ 3.0 and ϕ 4.5. The mechanical properties of steel bars were tested using an axial tensile test, according to the standard [32]. The mean value of the yield strength *f_y_* and the tensile strength *f_R_* for steel bars ϕ = 3.0 amounted to *f_y_* = 161.7 MPa, *f_R_* = 278.2 MPa, whereas for steel bars ϕ = 4.5, they amounted to *f_y_* = 274.5 MPa, *f_R_* = 398.9 MPa. The distribution of reinforcement is shown in Figure 2. The description of the beams and properties of concrete and steel are presented in Table 1.

**Figure 3:**
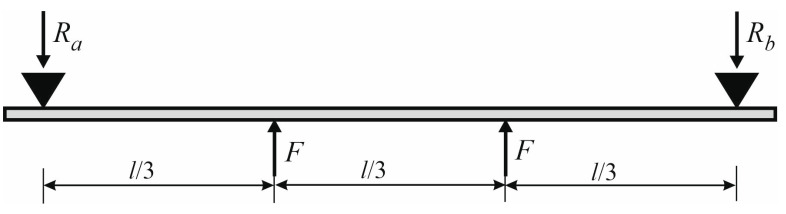
The scheme of loading.

The test stand was designed in such a way, that it was possible to observe the work of the beam in the post-critical range. Loads were imposed from bottom to top by two forces (the static scheme is shown in Figure 3). The method of loading consisting of forcing displacements was used.

**Figure 4:**
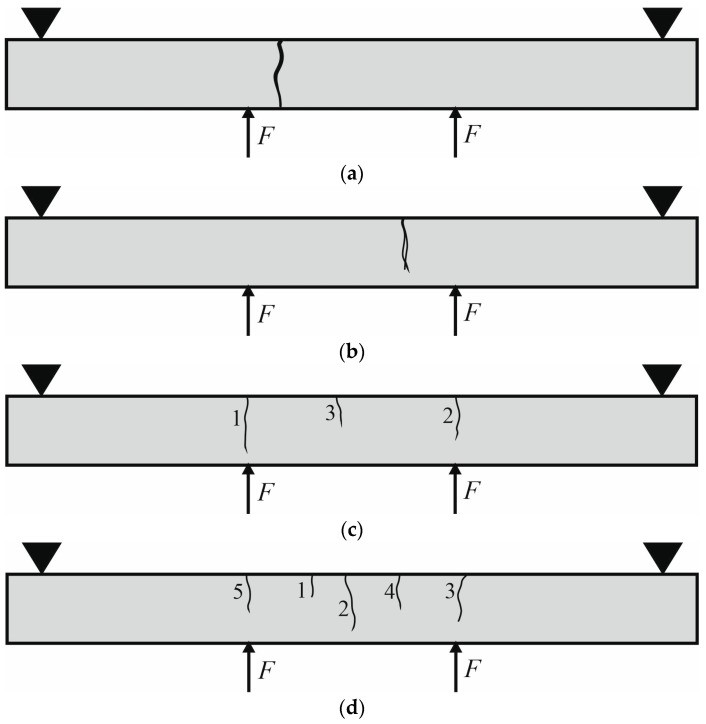
Cracks’ distribution in beams: (**a**) Beam No. 2; (**b**) Beam No. 5; (**c**) Beam No. 10; (**d**) Beam No. 13.

Changing failure modes in beams with different reinforcement ratios were observed during the experimental investigation. The examples of cracks’ distributions in the beams are presented in Figure 4. Concrete beams without reinforcement showed a rapid development of failure crack (see an example in Figure 4a). Although one crack appeared in beams with low reinforcement ratios of 0.05%, 0.07% and 0.09%, a less brittle character of failure was noticed, and the crack did not propagate through the whole section (see an example in Figure 4b). A more stable process of failure was observed in beams with reinforcement ratios of 0.12% and 0.20%. Three cracks developed when *ρ* = 0.12% (see an example in Figure 4c), and five cracks developed when *ρ* = 0.20% (see an example in Figure 4d).

In all tested beams with low reinforcement, the crack resistance was higher than the cracking moment measured in plain concrete beams. Cracking moments and maximum measured bending moments obtained in tested beams are presented in Table 2.

The analysis of the obtained results leads to the conclusion that the load-carrying capacity of lightly reinforced concrete members is determined by the cracking moment. In concrete beams and beams with low reinforcement ratios of 0.05%, 0.07% and 0.09%, the cracking moment was the maximum measured bending moment. In beams with a reinforcement ratio of 0.12%, an insignificant increase was noted in load capacity over the cracking moment, whereas in the beam with a reinforcement ratio of 0.20%, this increase reached 33%. Simultaneously, it was noticed that the crack resistance in beams with low reinforcement ratios was higher than the cracking moment in unreinforced concrete beams.

In order to explain the increase in the crack resistance of members with low reinforcement compared to non-reinforced ones, numerical calculations were performed. Additionally, finite element method (FEM) simulations were employed to analyze the influence of the selected steel bars on the load capacity of bending members.

### 2.2. Numerical Calculation

When modeling the cracking processes in concrete structures, non-linear fracture mechanics is applied, and among concrete crack models, the microcrack band model has frequently been chosen, for example, by Bažant and Oh [33], Bosco and Carpinteri [34], Carpinteri [35]. This model was applied for the analysis of crack resistance of lightly reinforced concrete beams.

**Figure 5:**
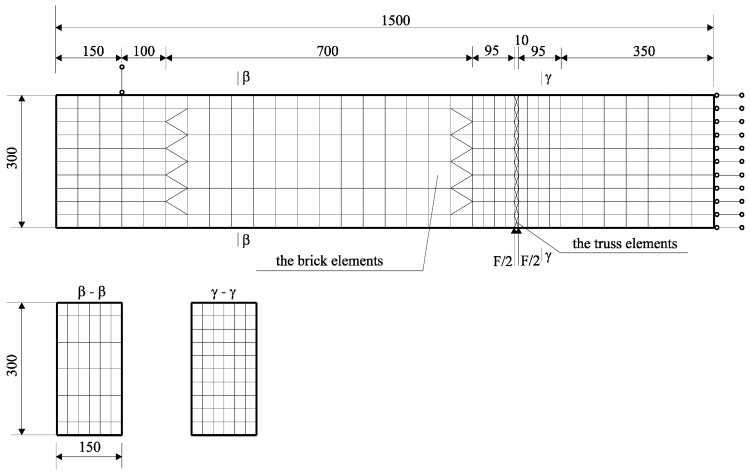
The FEM mesh of the analyzed beam (dimensions in millimeters).

**Figure 6:**
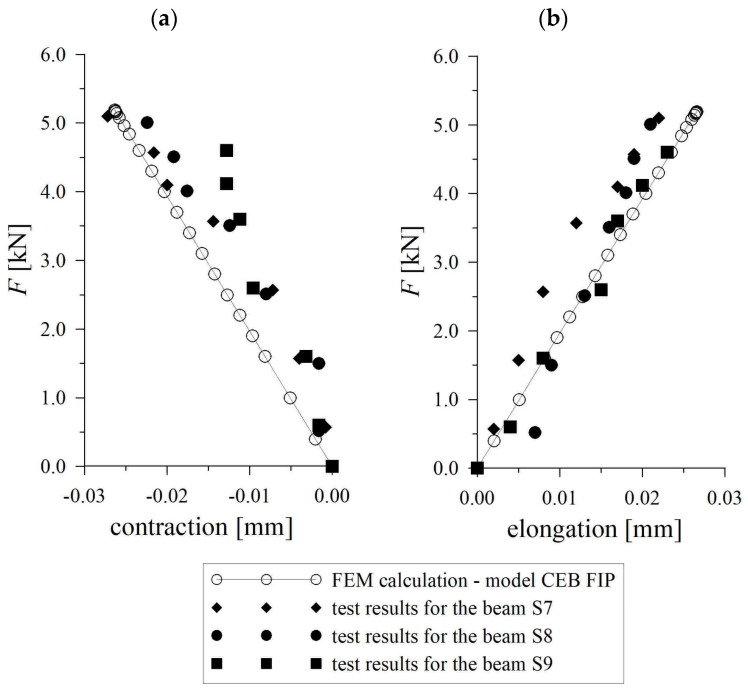
Comparison of numerical results with test results (**a**) in the compression zone; (**b**) in the tension zone.

The calculations were performed using a commercial finite element program. The beam model was composed of brick and truss elements. The non-linear characteristic of tensile concrete was applied in the fracture zone, which was modeled in the section with the highest bending moment values (the section where the load was put). The calculations employed the softening characteristic for tensile concrete given in the CEB-FIP Model Code [12]. In the CEB-FIP model of tensile concrete, the stress–crack opening relation in the post-critical range is described by a descending curve, and therefore, the modified Newton method with line search was used during FEM calculations. Several iterations were needed to obtain reliable results. Furthermore, the choice of the width of the fracture process zone was of crucial importance. The fracture process zone width *w_c_* was modeled to amount to 10 mm. The positive verification of the chosen value *w_c_* = 10 mm was presented in Ref. [36]. Outside the fracture process zone, brick elements were used for modeling the concrete as an elastic material. Taking into account the beam’s symmetry, only half of the beam was modeled, as presented in Figure 5. The finite element mesh was thickened in the vicinity of the modeled fracture process zone and in the support region where the stress was concentrated. The comparison of numerical results with test results showed high compatibility (as presented below; see Figure 6). The correctness of the selected FEM mesh was analyzed, and it was found to be sufficiently satisfactory for describing the phenomenon examined [37].

During FEM simulation, the concrete beam and beams with different reinforcement ratios were subjected to calculations. The ratio of steel bar distribution was used—as in the experiment—and additional cases were modeled in order to analyze the influence of steel bar distribution on crack resistance. For example, two beams with the same reinforcement ratio of 0.12% were considered but with bars with different diameters and numbers 3 ϕ 4.5 mm and 5 ϕ 3.5 mm provided.

High correlation was noticed between the experimental results and numerical calculations. For example, the cracking moment calculated for concrete beam *M_cr,FEM_* = 5.18 kNm was approximate to the mean value of the cracking moment in experiment *M_cr,E_* = 5.08 kNm. Significant correlation was also observed when comparing the curves of deflection versus applied load, which were reached during the experiment, with those obtained on the basis of numerical calculations (see Figure 6). The results presented in Figure 6 are related to deflections, which were measured during the experiment, and corresponding calculated FEM results for plain concrete beams in the compression and tension zone.

**Figure 7:**
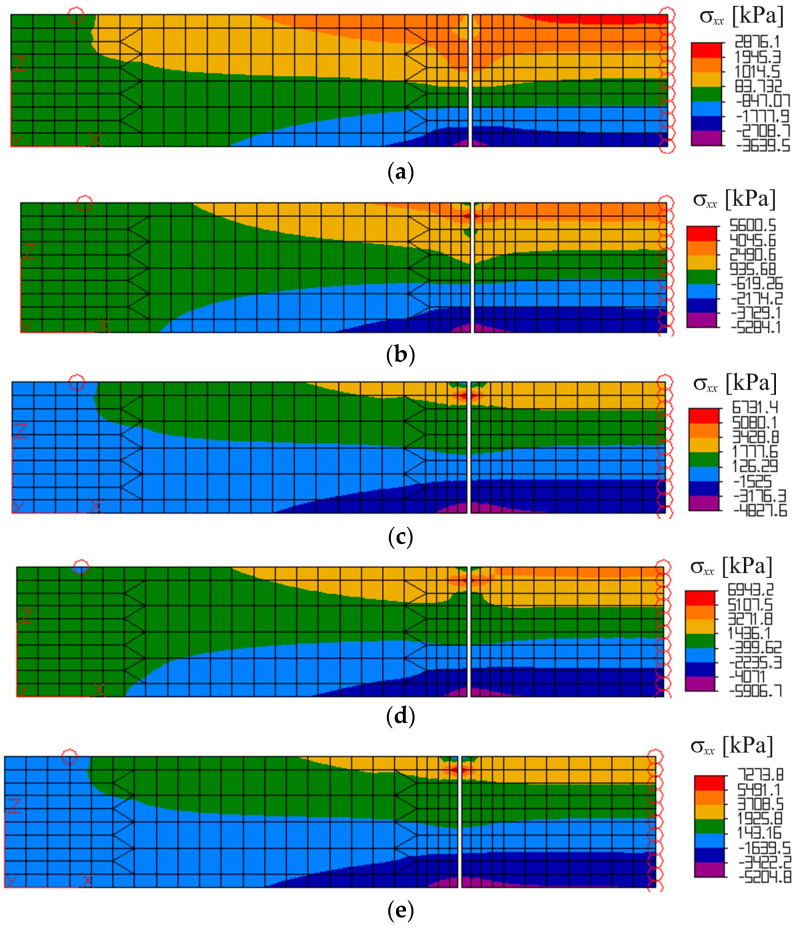
FEM results for beams: (**a**) Concrete beam; (**b**) Beam with bars 3 ϕ 3.5 mm; (**c**) Beam with bars 3 ϕ 4.5 mm; (**d**) Beam with bars 5 ϕ 4.5 mm; (**e**) Beam with bars 3 ϕ 6.0 mm.

The results obtained from FEM calculation were analyzed. The influence of reinforcement was noticed when watching a strain and stress development in the beams in the following calculation steps. The visualization of stress distribution σ_xx_ in all the simulated beams is presented in Figure 7. The most significant difference in deformation was observed in beams in the vicinity of the fracture process zone depending on the reinforcement applied.

**Figure 8:**
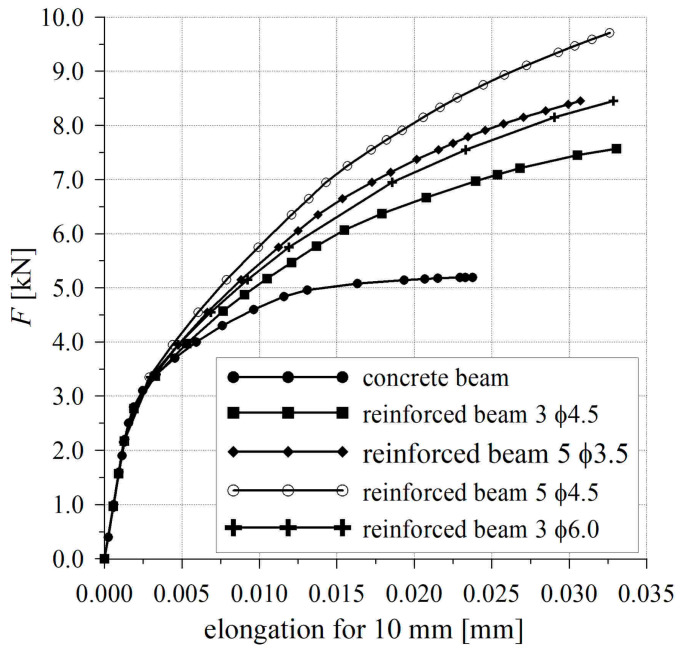
The comparison of elongation in the fracture process zone for all calculated beams.

The influence of the reinforcing bars provided was analyzed in depth in the fracture process zone, where the difference in crack development was noticed. The comparison of the force–elongation curves in the fracture process zone is presented in Figure 8. It was observed that with the increase in the reinforcement ratio, the maximum force and the ultimate elongation increased. The influence of the distribution of steel bars on crack resistance was observed as well.

**Figure 9:**
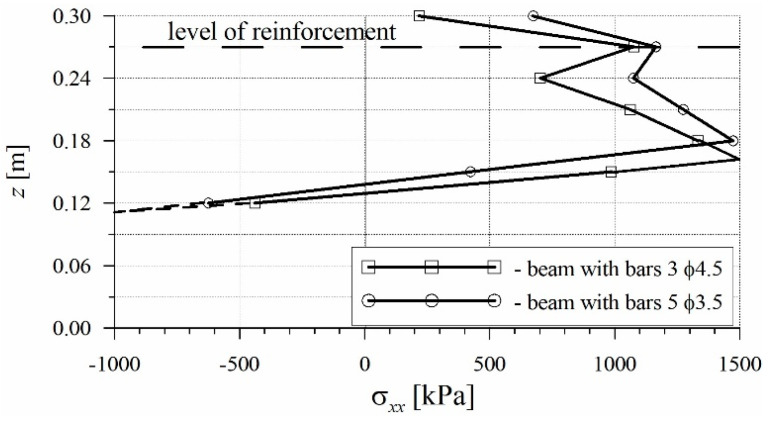
Diagrams of normal stress in the fracture process zone at the same load stage for the beam *ρ* = 0.12% with steel bars 3 ϕ 4.5 and 5 ϕ 3.5.

The calculation results were compared for two beams in which the cross-section of reinforcement was identical, and the difference consisted of the selection of steel bars: 3 ϕ 4.5 mm and 5 ϕ 3.5 mm. Higher crack resistance was obtained in the beam with a larger number of bars and a smaller diameter 5 ϕ 3.5 mm. In order to describe this phenomenon, the stress distribution was analyzed in the fracture zone in the section through the modeled crack. In Figure 9, the stress distributions of the calculated normal stress *σ_xx_* are juxtaposed at the same load level for two beams: those with steel bars 3 ϕ 4.5 mm and 5 ϕ 3.5 mm. The axis oriented along the height of the beam’s cross-section is described in Figure 9 as *z* in meters. Because of the reversed static scheme, which was applied during the testing of beams (the same was employed in the numerical simulation), the tension zone is situated in the upper part of the beam. In Figure 9, the location of the steel bars is indicated. It can be observed that the chance of stress distribution in the tension zone obtained from numerical simulation was caused by the presence of steel bars, and it was most visible at the level of reinforcement.

It was found that the selection of reinforcing bars influenced the process of crack formation. In the beam with reinforcement of 5 ϕ 3.5 mm, a smaller decrease in stress is observed, which proves that the process of crack formation is slower than in the beam with reinforcement of 3 ϕ 4.5 mm. This is caused by a more uniform distribution of tensile stress in the concrete surrounding the steel bars and in the concrete between the bars. A more effective cooperation between concrete and reinforcement can be obtained when the total contact area between the bars and concrete is larger, as in the case of reinforcement with a larger number of bars.

The FEM analysis conducted indicated that, apart from the reinforcement ratio, the crack formation and distribution of normal stresses in the beam are also influenced by the selection of reinforcing bars.

## 3. Results and Discussion

To quantitatively describe the influence of reinforcement on the crack resistance of beams, the results of our own research were juxtaposed with those obtained by Dąbrowski [15]. On their basis, we calculated the ratio of the cracking moment in a lightly reinforced concrete member *M^SRC^* to the cracking moment in the member without reinforcement *M_cr_*. The *M^SRC^*/*M_cr_* ratio describes the increase in crack resistance. The second parameter, defined as the reinforcement dispersion ratio *i_s_*, was established in order to include the distribution of steel bars in the analysis of crack resistance. The parameter is described by the formula

(4) $$i_{s}=\rho \frac{\delta }{\varphi },$$

where *ρ*—reinforcement ratio; *φ*—steel diameter in m, *δ* = 10^−3^ m.

**Figure 10:**
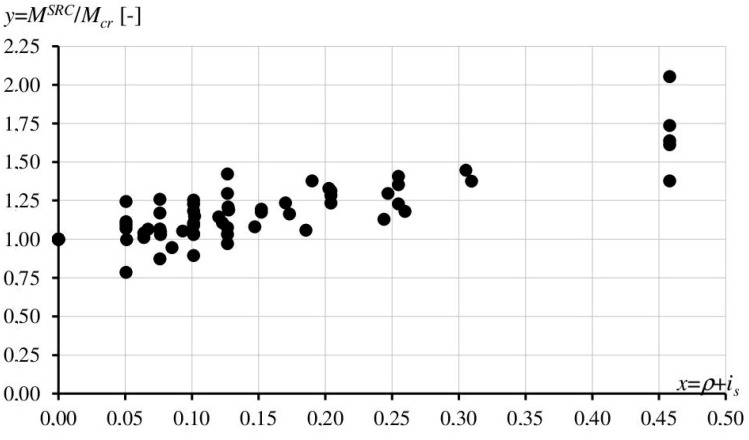
Crack resistance *y* = *M^SRC^*/*M_cr_* [-] versus reinforcement *x* = *ρ* + *i_s_* [%]—Experimental results.

On the basis of our own experimental results and the test results reported in Ref. [15], the set of experimental data was obtained. The crack resistance *y* = *M^SRC^*/*M_cr_* was subordinated with the coefficient *x*, which is the sum of the reinforcement ratio *ρ* and the coefficient of reinforcement dispersion *i_s_* (*x* = *ρ* + *i_s_* expressed in %). The relation between *y* = *M^SRC^*/*M_cr_* and coefficient *x* is presented in Figure 10.

**Figure 11:**
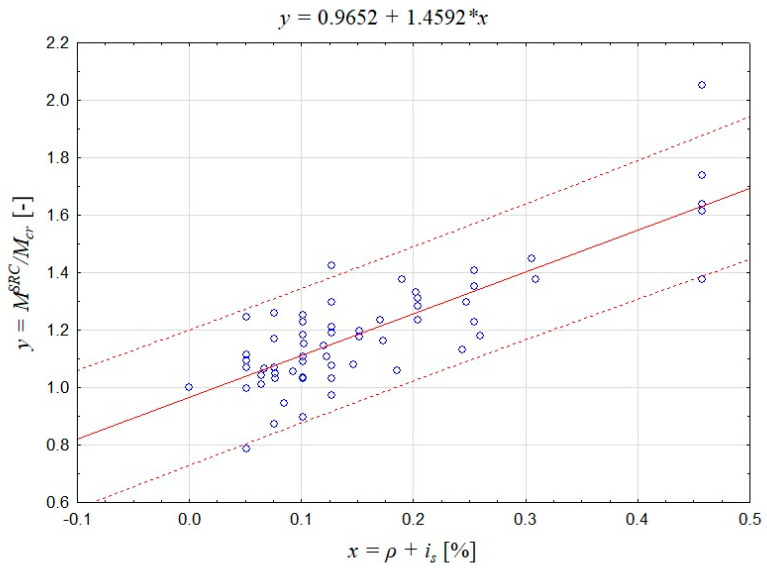
Regression model of the increase in crack resistance *y* = *M^SRC^*/*M_cr_* [-] depending on the reinforcement ratio and dispersion *x* = *ρ* + *i_s_* [%].

A statistical analysis was performed on the experimental results shown in Figure 6. Based on the approximation of the results, the regression model was evaluated via linear regression using the reinforcement *x* = *ρ* + *i_s_* as the predictor variable and the increase in crack resistance *y* = *M^SRC^*/*M_cr_* as the response variable (the results of the regression analysis are presented in Table 3 and in Figure 11). The correlation coefficient R was 0.8335, which indicated a fairly strong linear relationship between the predictor *ρ* + *i_s_* and the response variable *M^SRC^*/*M_cr_*. The regression model describes the relation between the crack resistance of lightly reinforced concrete member and the reinforcement provided, which shows high correspondence with the test results. The coefficient of determination reached R^2^ = 0.6947, and the mean absolute percentage error amounted to MAPE = 7.11%.

The high correlation between the test results and the regression model confirms the correctness of the concept of taking into account the reinforcement dispersion ratio together with the reinforcement ratio when determining the crack resistance of lightly reinforced concrete members. Based on the regression model obtained, a formula was proposed for determining the flexural capacity of lightly reinforced concrete members:

(5) $$M_{ult}^{SRC}=M^{SRC}=W_{c}\kappa f_{ctm}\left[1.0+146\left(\rho +i_{s}\right)\right],$$

## 4. Conclusions

In the study performed, an increase was obtained in the crack resistance of concrete members with low reinforcement ratios in comparison with the cracking moment in concrete elements. It was found that the cracking moment in the beams tested depended on the reinforcement ratio and steel bar distribution. Furthermore, the numerical simulation provided an insight into the effect of steel bars on stress distribution in the fracture process zone.

A method was proposed for estimating the crack resistance of lightly reinforced concrete members. Both the reinforcement ratio and the reinforcement dispersion ratio were taken into consideration. Furthermore, the coefficient *κ* expressing the ratio *f_ctf_*/*f_ctm_* was used in the proposed formula (Equation (5)), which ensured that the scale effect and the influence of concrete fracture properties on crack resistance could be included.

The proposed method provides the basis for extrapolation of the test results obtained for small elements and conclusions for members with larger cross-sections, such as foundations, which frequently use lightly reinforced concrete.

## Figures and Tables

**Table 1:** Beams tested and properties of concrete and steel.

Member’s Number	Steel Bars ϕ [mm]	Reinforcement Ratio *ρ* [%]	Concrete Properties				Steel Properties	
			*f_cm_* [MPa]	*f_ctm_* [MPa]	*E_cm_* [GPa]	*G_F_* [Nm/m^2^]	*f_y_* [MPa]	*f_R_* [MPa]
1; 2; 3	-	0	20.5	1.5	22.1	82.9	-	-
4; 5	3 ϕ 3.0	0.05	24.5	2.4	23.3	93.6	161.7	278.2
6; 7	4 ϕ 3.0	0.07	24.5	2.4	23.3	93.6	161.7	278.2
8; 9	5 ϕ 3.0	0.09	24.5	2.4	23.3	93.6	161.7	278.2
10; 11	3 ϕ 4.5	0.12	20.5	1.5	22.1	82.9	274.5	398.9
12; 13	5 ϕ 4.5	0.20	20.5	1.5	22.1	82.9	274.5	398.9

**Table 2:** Experimental cracking moments and maximum bending moments in tested beams.

Member’s Number	Steel Bars ϕ [mm]	Reinforcement Ratio *ρ* [%]	Experimental Cracking Moment *M_cr,E_* [kNm]	Maximum Bending Moment *M_max_* [kNm]	*M_max_*/*M_cr,E_* [-]
1	-	0	4.639	4.639	1
2		0	5.473	5.473	1
3		0	4.654	4.654	1.00
4	3 ϕ 3.0	0.05	6.835	6.835	1.00
5	3 ϕ 3.0	0.05	6.891	6.891	1.00
6	4 ϕ 3.0	0.07	7.212	7.212	1.00
7	4 ϕ 3.0	0.07	6.364	6.364	1.00
8	5 ϕ 3.0	0.09	7.038	7.038	1.00
9	5 ϕ 3.0	0.09	7.709	7.709	1.00
10	3 ϕ 4.5	0.12	5.528	5.978	1.08
11	3 ϕ 4.5	0.12	5.364	5.724	1.07
12	3 ϕ 4.5	0.12	5.092	5.353	1.05
13	5 ϕ 4.5	0.20	5.538	7.378	1.33

**Table 3:** Summary of the regression analysis.

Observations N = 69	Regression Summary for Dependent Variable *y* = *M^SRC^*/*M_cr_*: Correlation Coefficient R = 0.83351040; Coefficient of Determination R^2^ = 0.69473060; Adjusted R^2^ = 0.69018247					
	b*	Standard Error of b*	*b*	Standard Error of *b*	t Stat (67)	*p*-Value
Intercept			0.965170	0.021558	44.77145	0.00000
*x* = *ρ + i_s_* [%]	0.833510	0.067499	1.459224	0.118170	12.34847	0.00000

## Data Availability

The data supporting the findings of this study are available on request from the corresponding author.
